# Peaks in bat activity at turbines and the implications for mitigating the impact of wind energy developments on bats

**DOI:** 10.1038/s41598-021-82014-9

**Published:** 2021-02-11

**Authors:** Suzanne M. Richardson, Paul R. Lintott, David J. Hosken, Theo Economou, Fiona Mathews

**Affiliations:** 1 Hatherly Laboratories, Biosciences, College of Life and Environmental Sciences, University of Exeter, Prince of Wales Road, Exeter, EX4 4PS UK; 2 Univerity of the West of England, Coldharbour Lane, Bristol, BS16 1QY UK; 3 Centre for Ecology & Conservation, University of Exeter, Cornwall, Tremough, Penryn, Cornwall, TR10 9EZ UK; 4 Harrison Building, College of Engineering, Mathematics, and Physical Sciences, University of Exeter, N Park Rd, Exeter, EX4 4QF UK; 5 University of Sussex, John Maynard Smith Building, Falmer, Sussex, BN1 9QG UK

**Keywords:** Ecology, Biodiversity, Conservation biology

## Abstract

Wind turbines are a relatively new threat to bats, causing mortalities worldwide. Reducing these fatalities is essential to ensure that the global increase in wind-energy facilities can occur with minimal impact on bat populations. Although individual bats have been observed approaching wind turbines, and fatalities frequently reported, it is unclear whether bats are actively attracted to, indifferent to, or repelled by, the turbines at large wind-energy installations. In this study, we assessed bat activity at paired turbine and control locations at 23 British wind farms. The research focussed on *Pipistrellus* species, which were by far the most abundant bats recorded at these sites. *P. pipistrellus* activity was 37% higher at turbines than at control locations, whereas *P. pygmaeus* activity was consistent with no attraction or repulsion by turbines. Given that more than 50% of bat fatalities in Europe are *P. pipistrellus*, these findings help explain why Environmental Impact Assessments conducted before the installation of turbines are poor predictors of actual fatality rates. They also suggest that operational mitigation (minimising blade rotation in periods of high collision risk) is likely to be the most effective way to reduce collisions because the presence of turbines alters bat activity.

## Introduction

The number of wind turbines is rapidly increasing globally as the demand for renewable energy grows^1^. While wind power plays a vital role in reducing carbon emissions; it also has negative consequences for the environment. These include noise and visual pollution^2^, habitat fragmentation and wildlife displacement^3^ and direct collision risk for bats and birds^4,5^. Wind farms negatively affect over 30 bat species^4^ and have potential consequences for population viability of at least one species^6^. Given the projected increase in wind power^1^, it is crucial to consider these negative ecological effects carefully.

Despite over a decade of research on bat fatalities at wind farms, relatively little is known about why wind turbines kill bats. Observations using infra-red imagery^7^ and satellite tagging^8^ have both determined that bats appear to interact with turbines, which may heighten collision risk. Roeleke et al.^8^ found that female *Nyctalus noctula* repeatedly came into close contact with wind turbines during foraging flights and flew at heights that suggested a high risk of colliding with turbine blades. Bats interact with turbines and changing their orientation when approaching them depending on wind speed^9^. For tree-roosting bats, this may be a consequence of individuals mistaking turbines for potential roosting sites or bats attempting to establish mating sites at turbines^9^. However, this is unlikely to be the primary explanation in Europe, where most casualties are not tree-roosting species. Increased prey availability around turbines is also a hypothesis for increased bat presence around turbines^9,10^. Although evidence suggests bats are attracted to large turbines, experimental assessment at small wind turbines found that bats actively avoided them^3,11,12^.

Recently, we showed that pre-construction acoustic surveys, which form part of Environmental Impact Assessments, are poor predictors of bat casualties at wind farms^13^. Understanding whether this is because bats are actively attracted to turbines is fundamental in designing appropriate risk assessments, as pre-construction surveys are not good indicators of collision risk if turbines themselves are attractive features. Here, we conducted a paired observational study across 23 wind farms in Britain to assess whether bat activity is higher at turbines compared to controls. The control locations were of comparable habitat, elevation, and land management, whilst being as far away from wind turbines as possible while still being within the boundary of land managed by the same landowner. We hypothesised that there would be no difference between bat activity (assemblage and frequency of echolocation passes) at turbines and paired controls. We predicted that if bats were attracted to turbines, then the activity would be higher at turbines than controls.

## Results

**Figure 1:**
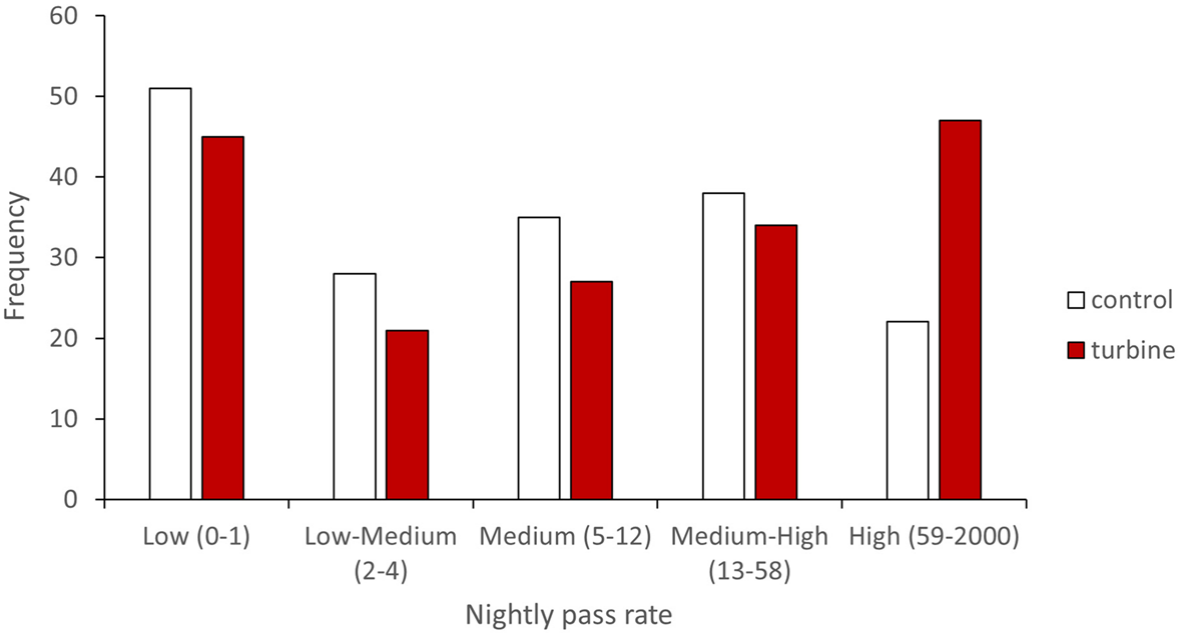
Frequency of nights of *P. pipistrellus* activity which can be classed at either low (1st–20th percentile), low–medium (21st–40th percentile), medium (41st–60th percentile), medium–high (61st–80th percentile), or high (81st–100th percentile) activity levels split between turbine and control locations.

During 433 paired detector nights, we recorded 43,196 bat calls, of which two *Pipistrellus* species accounted for 91% of all activity (*P. pipistrellus* 66%, and *P. pygmaeus* 25%). The proportion of *P. pipistrellus* calls was higher at the turbine (74% of all calls) compared to the control (47%); whereas the proportion of *P. pygmaeus* calls was higher at the control (38%) compared to the turbine (19%). We recorded 30,102 (70%) bat passes at turbines and 13,094 (30%) at controls. Of the 22 sites which recorded *P. pipistrellus*, 16 sites (73%) had higher average nightly bat activity at the turbine compared to the control location. Similarly, of the 18 wind farms which recorded *P. pygmaeus*, 12 sites (67%) had higher average nightly activity at the turbine compared to the control location. Nights of high *P. pipistrellus* activity (81st–100th percentile following Lintott et al.^14^) are predominantly at the turbine (68% of occasions) rather than the control (Fig. 1). In contrast, nights of high *P. pygmaeus* activity are split evenly between turbines (53%) and control (47%) locations.

**Figure 2:**
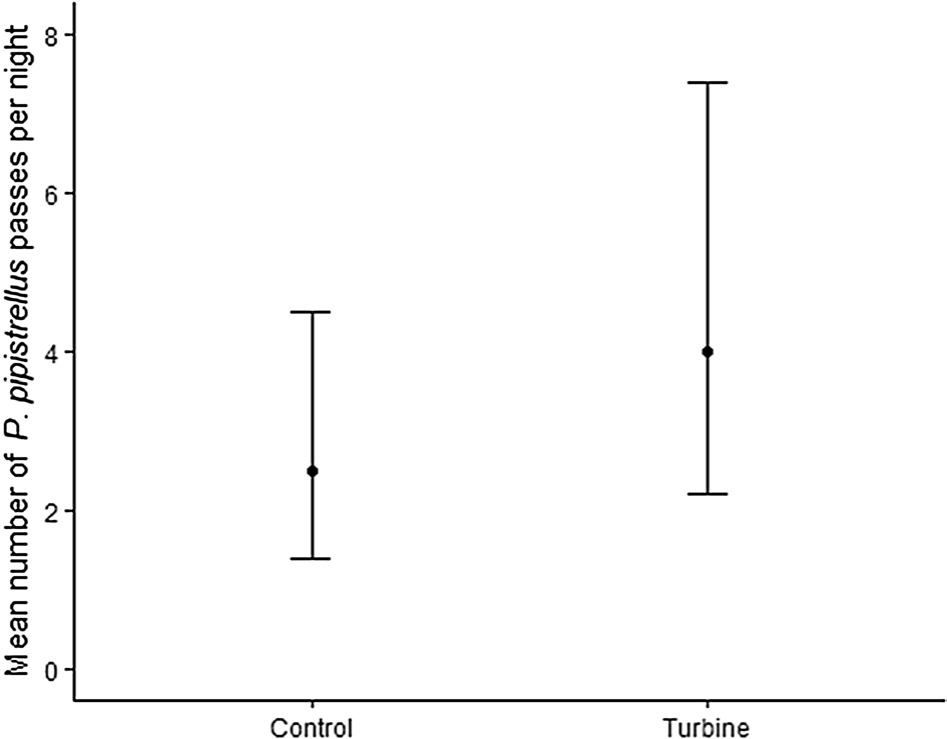
Predicted mean passes per night with 95% confidence intervals at the turbine and control locations across 23 wind farm sites for *P. pipistrellus*.

The degree to which activity differed at turbine and control locations varied between species. Based on the estimated coefficients from the GLMM (Table 1), activity (reported as predicted mean passes per night) for *P. pipistrellus* was 37% higher at turbines (4 95% CI 2–7) compared to controls (3 95% CI 1–5; Fig. 2). This difference was statistically significant when we restricted analyses to only those nights with activity (activity nights) at either the turbine (4 95% CI 2–7) or the control (2 95% CI 1–5). For *P. pygmaeus*, there was no significant difference between the turbine (0.2 95% CI 0–1) and the control (0.1 95% CI 0–0.4), for all nights, and activity nights (turbine: 3 95% CI 2–5, control: 2 95% CI 1–4).

**Table 1:** Summary of negative binomial mixed models to assess the difference in *P. pipistrellus* and *P. pygmaeus* activity at the turbine and paired control locations at 23 British wind farms.

Species	Including nights with no activity at either of pair	Estimate ± SE	Log-likelihood	Χ^2^	df	*P* value
*P. pipistrellus*	Yes	0.42 ± 0.15	− 2473	7.59	1	**0.006**
*P. pipistrellus*	No	0.47 ± 0.17	− 2266	7.46	1	**0.006**
*P. pygmaeus*	Yes	0.24 ± 0.21	− 1734	1.34	1	0.248
*P. pygmaeus*	No	0.21 ± 0.18	− 1488	1.42	1	0.234

A positive estimate indicates higher levels of activity at the turbine compared to the control.

## Discussion

Wind energy production is undergoing a rapid global expansion which helps combat greenhouse gas emissions but also poses conservation risks to some taxa. Here, we show in a study replicated across multiple wind farms, that *P. pipistrellus* has higher levels of activity at wind turbines than at paired control locations. Given that *P. pipistrellus* is one of the most abundant bats in Europe, and one which incurs the highest fatalities at wind farms across Europe^15^, the findings are essential in the planning of future developments. The observed higher levels of activity could be because there are more bats around turbines, or because animals spend more time in these locations relative to controls, even if the number of individual bats remains the same. We cannot distinguish between these possibilities using acoustic data. However, either way, higher levels of activity around turbines is likely to increase fatality risks and help to explain why fatality rates are often not predicted by acoustic surveys for bat activity conducted prior to facility construction^13^.

These results contrast with Barré et al.^16^, who found a significant negative effect of proximity to turbines on the activity of *P. pipistrellus*. The differences in results might reflect the positioning of the bat detector used as the control. Barré et al.^16^ positioned their detectors alongside hedgerows, linear features which are used preferentially by *P. pipistrellus*^17^. In contrast, we placed control detectors in open habitats comparable to the placement of wind turbines—Eurobats Guidelines advise against the placement of turbines close to linear features. Our results support Cryan et al.^9^ who observed that individual bats are attracted to turbines.

In this study, the mean number of bat passes per night at turbines and controls was low because typically wind farms are sited in habitats considered to be largely unsuitable for bats. However, activity was highly variable across nights, with a maximum pass rate of 2,173 passes per night. On nights with high activity, a 37% difference in activity between the turbine and control could be biologically meaningful. Although higher levels of activity around turbines is possible for both *Pipistrellus* species, the strength of the association was higher for *P. pipistrellus,* suggesting that species-specific differences may exist.

Detectors placed at 2 m high will typically pick up calls of *Pipistrellus spp*. up to 30 m away. However, this distance can sometimes be further, depending on the orientation of the bat, weather conditions, and call volume. For 15 of our 23 sites, the distance between the ground and the blade tip was 30 m or less, and at 20 of the 23 sites was 40 m or less. Even if bats were foraging closer to the ground, they would still be at risk of collision with the blade tips near the ground. *Pipistrellus* species commonly fly between 5 and 10 m^17^, hence it is unlikely that many bat passes from this genus would be missed at control locations. While monitoring at 2 m would miss some calls in the rotor sweep area, conversely monitoring from the nacelle would also miss calls, given the blade diameters were up to 100 m. To investigate whether any vertical displacement of bat activity occurs in response to the presence of turbine blades, we recommend the use of arrays of acoustic detectors or imaging systems to permit precise triangulation of bat locations^18^.

Although operational mitigation is becoming increasingly frequent (i.e. feathering wind turbine blades during periods of high collision risk for bats^19^), there are many countries which still rely heavily on pre-construction surveys to determine planning permission decisions for wind farms^20,21^. Lintott et al.^13^ found that bat activity before turbine construction was a poor predictor of the number of fatalities at wind farms. However, it was unclear if this was a consequence of insufficient pre-construction survey effort, ineffective mitigation strategies, or because of bat behaviour changes after the construction of turbines. Our results support the latter proposition, and therefore conducting more extensive and costly pre-construction surveys is unlikely to provide better estimates of fatality risk, since the behaviour of a key species differs in response to the presence of a turbine.

In summary, in a broad survey across the UK, we found evidence that at least one common bat species had higher activity at wind turbines. Coupled with earlier work, this suggests assessing potential impacts of turbines on bats is not straight forward, and operation mitigation is likely to be vital in reducing turbine impacts on bat populations.

## Methods

### Survey design

Surveys were conducted at wind farms (defined herein as a site) in 2011 (five sites), 2012 (eight sites) and 2013 (ten sites) between July and October, to coincide with the peak fatality period identified in Europe^15^. We surveyed bat activity using broad-spectrum static acoustic detectors at sites that were part of the National Bats and Wind Turbines Project^22^ (NBWT). The NBWT project aimed to understand the risks to bat populations posed by wind turbine developments in Great Britain. The project investigated bat mortality and activity at a range of wind energy facilities, and the results were used to revise national guidance on the installation and operation of wind turbines. Here, we monitored sites for a mean of 19 (SD 9) nights. At each site, we randomly selected a single turbine and identified a control location to monitor acoustically. We made recordings from the ground with microphones placed on tripods at approximately 2 m high. We did not monitor at the nacelle of turbines because it was not possible to record bat activity at the same height at control locations. Bat passes were recorded from 30 min before sunset until 30 min after sunrise, using full-spectrum real-time acoustic recorders (SM2BAT and SM2BAT+, Wildlife Acoustics, MA, USA). Acoustic recorders were programmed to record at a sampling frequency of 192 kHz when triggered by a signal to noise threshold over 16 kHz that was also above 36 dB (year 1 and 2) or 48 dB (year 3), adjusted in-line with manufacturer recommendations. When triggered, recording continued until the signal to noise ratio dropped below the threshold for at least one second.

We determined the location of the control detector by the presence of habitat (Table 2), elevation and land management that was comparable to the selected turbine. We located the control detector as far away from the wind turbines as possible, while still being within the boundary of land managed by the same landowner. This selection process prevented turbines always being on the edge of a turbine cluster or the end of a line of turbines which can influence bat activity levels. Hence, the monitored turbine was not usually the closest turbine to the control. The controls were a mean distance of 673 m (SD 393, range 222 to 1939 m) from the nearest turbine and 1463 m (SD 1064, range 222 to 4150 m) from their paired turbine. We monitored controls at 23 of the 46 NBWT sites across Britain. We surveyed at a subset of sites as various factors (e.g. landowner permission, difficulties in locating suitable control habitats, and failure of detectors) prevented controls at all NBWT sites. We also excluded controls monitored as part of the NBWT that were located within 50 m of turbines and those located on linear features which were not comparable to the turbine location. The mean number of wind turbines within site was 18 (SD 14). Sites ranged from relatively small wind power facilities containing just six turbines up to relatively large sites containing 68 turbines. The mean tower height was 62 m (SD 19, range 35 to 102 m) and the mean distance between the ground and the blade was 27 m (SD 16, range 12 to 78 m).

**Table 2:** Summary of landscape metrics—the mean and SD for the minimum distance to broad habitats and percentage cover of broad habitats within 250 m and 2500 m of the paired turbine and control locations across 23 wind farm sites.

	Control	Turbine
Farmland min	213 m (401)	320 m (424)
Semi-natural habitat^1^ min	567 m (868)	702 m (908)
Total woodland min	532 m (387)	654 m (503)
Urban min^2^	98 m (100)	73 m (126)
Water riparian^3^ min	133 m (113)	164 m (116)
Farmland 250 m	53% (42)	37% (42)
Semi-natural habitat 250 m	9% (16)	12% (26)
Total woodland 250 m	2% (5)	4% (8)
Urban 250 m	2% (2)	2% (1)
Water riparian 250 m	7% (18)	3% (6)
Farmland 2500 m	45% (31)	38% (33)
Semi-natural habitat 2500 m	12% (12)	11% (11)
Total Woodland 2500 m	12% (12)	13% (12)
Urban 2500 m	2% (2)	2% (2)
Water riparian 2500 m	6% (11)	6% (9)

Habitat data were extracted from Land Cover Map 2007^30^; the urban statistics were extracted from OS Mastermap^31^.

^1^Rough grassland and scrub, ^2^buildings, structures, and roads, ^3^inland water, coastal water, bog, fen, marsh, and swamp.

### Species identification

Bat calls were processed using Kaleidoscope Pro (v.1.1.20, Wildlife Acoustics, Massachusetts, USA) with British bat classifiers (v.1.0.5). First wac files were converted to wav, separating trigger events creating individual files classified as potential bat files or noise files. The potential bat files are a continuous run of pulses not separated by a time gap of more than one second^23^. All potential bat files were manually analysed, classifying them to a species or, where this was not possible, to genus or group (in the case of Nyctaloid bats only). We used call parameters from Russ^24^ to identify species. Owing to the vast numbers of call files, we identified a maximum of two passes per species within each call file. Hence, within a file, the presence of three or more bat passes of the same species was recorded as two passes: this situation occurred in four per cent of all bat files.

### Statistical analyses

We undertook all analyses using R Studio^25^, and the packages lme4^26^ and ggplot2^27^. We built Generalised Linear Mixed Models (GLMMs) with a negative binomial distribution to assess the differences in bat activity between turbine and control locations. Our analyses focused on *P. pipistrellus* and *P. pygmaeus* since most bat casualties at wind farms in Europe are *Pipistrellu*s species^28^, and most activity at our study sites was of these species. We fitted the number of *P. pipistrellus* passes per night as the dependent variable and detector location (a factor with two levels: control and turbine) as a fixed effect. We fitted site and an observation level factor (a unique factor for each detector night) as random effects, to account respectively for repeated nights of observation within site, and other unobserved effects that might influence activity^29^. We first ran models including nights which had no activity at both the control and the paired turbine included, and subsequently, re-ran them excluding these nights. We repeated these models with the same model structure, and the total number of *P. pygmaeus* passes per night as the dependent variable. We assess significance using log-likelihood ratio tests of the full model compared to the alternative model, which excluded the fixed effect (detector location). We evaluated model fit by checking for the normality of standardised residuals and that there was no evidence of heteroscedasticity.

## Data Availability

The dataset and R code generated and analysed during the current study are available from Figshare and the University of Sussex Research Data Repository 10.25377/sussex.13606802.
